# Supplementary material for: Self-powered triboelectric-responsive microneedles with controllable release of optogenetically engineered extracellular vesicles for intervertebral disc degeneration repair
Source: Nat Commun. 2024 Jul 9;15:5736. doi: 10.1038/s41467-024-50045-1 (PMC11233569; doi:10.1038/s41467-024-50045-1)
Supplement: Supplementary file 1 — Supplementary Information [file 41467_2024_50045_MOESM1_ESM.pdf]

Self-powered triboelectric-responsive microneedles with controllable release of optogenetically engineered extracellular vesicles for intervertebral disc degeneration repair

Weifeng Zhang, Xuan Qin, Gaocai Li, Xingyu Zhou, Hongyang Li, Di Wu, Yu Song, Kangcheng Zhao, Kun Wang, Xiaobo Feng, Lei Tan, Bingjin Wang, Xuhui Sun, Zhen Wen, Cao Yang

Supplementary Fig. 1. Exercise accelerated the degenerative process of IVDs from exercise-induced surgical lumbar instability.

Supplementary Fig. 2. Inflammatory response mediated the degenerative process of IVDs from exercise-induced surgical lumbar instability.

Supplementary Fig. 3. Senescent NP cells harbored STING-mediated inflammatory response and disassembly of TRAM1-TREX1 complex.

Supplementary Fig. 4. Loss of TRAM1 disrupted the tethering of TREX1 in the ER, and triggered cGAS-STING axis-mediated NP cell senescence.

Supplementary Fig. 5. Upregulation of TRAM1 in senescent NP cells reconstructed TREX1 function and alleviated cGAS-STING axis activation.

Supplementary Fig. 6. TRAM1-loading EXPLOR-engineered EVs alleviated inflammatory senescence phenotype acquisition of NP cells.

Supplementary Fig. 7. Structural design and electrical output performance of TENG.

Supplementary Fig. 8. The design and characterization of the triboelectric-responsive MNs with controllable release of EXPLOR-engineered EVs.

Supplementary Fig. 9. The experimental procedure of the ability of MNs to insert pig skin.

Supplementary Fig. 10. Triboelectric-responsive MNs delivered TRAM1-loading engineered EVs and mitigated inflammatory senescence of NP cells in vitro.

Supplementary Fig. 11 Illumination-induced TRAM1 loading into EVs was essential for the therapeutic effects of engineered EVs for remodeling TREX1 function and inhibiting NP cell senescent phenotypic acquisition.

Supplementary Fig. 12 EV-carrying MN systems didn't contribute to unexpected inflammatory response in the inserted skin and paravertebral tissues.

Supplementary Fig. 13 Self-powered triboelectric-responsive MN system alleviated the senescence and inflammatory response of NP cells in rat coccygeal IVDD.

Supplementary Fig. 14 Illumination-induced TRAM1 loading into EVs was essential for the therapeutic effects of engineered-EV carrying MN system for alleviating needle puncture-induced rat coccygeal IVDD.

Supplementary Fig. 15 The therapeutic efficacy of different delivery strategies for alleviating rat coccygeal IVDD.

Supplementary Fig. 1. Exercise accelerated the degenerative process of IVDs from exercise-induced surgical lumbar instability.

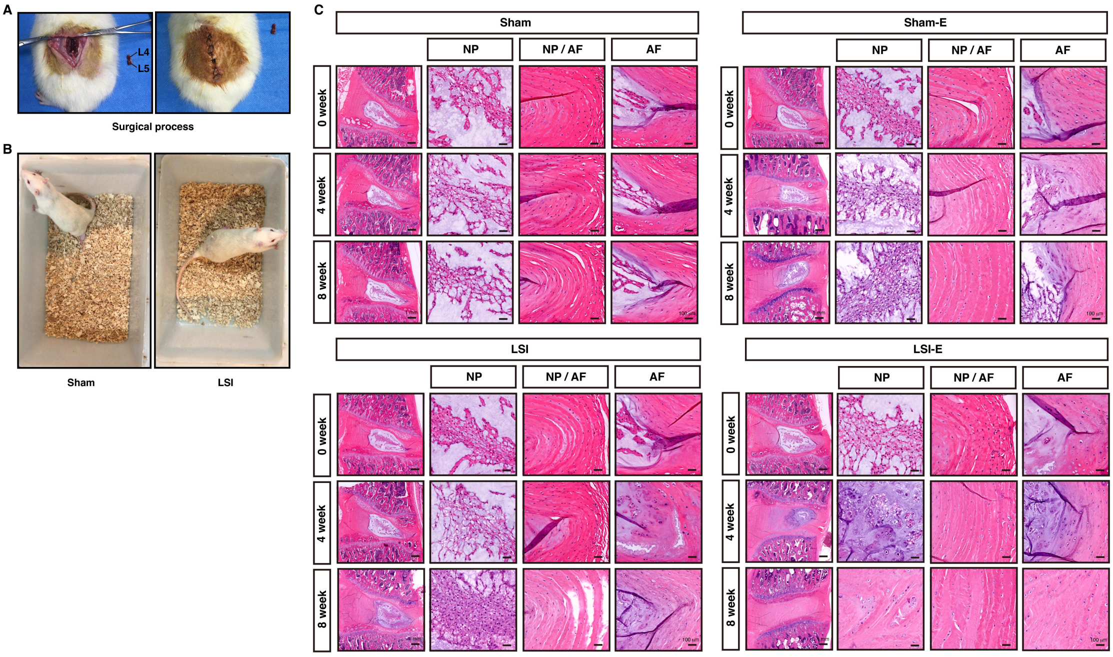

A) Surgical operation of resecting L4-L5 spinous processes of rat spine along with the supraspinous and interspinous ligaments to induce LSI. B) Movement posture pictures of rats from LSI or Sham operation in feeding cage. C) H&E staining of L4-L5 IVDs from LSI group with or without exercise intervention for 0, 4, and 8 weeks, bar: 1 mm, 100 μm (Representative image of five independent experimental replicates).

Supplementary Fig. 2. Inflammatory response mediated the degenerative process of IVDs from exercise-induced surgical lumbar instability.

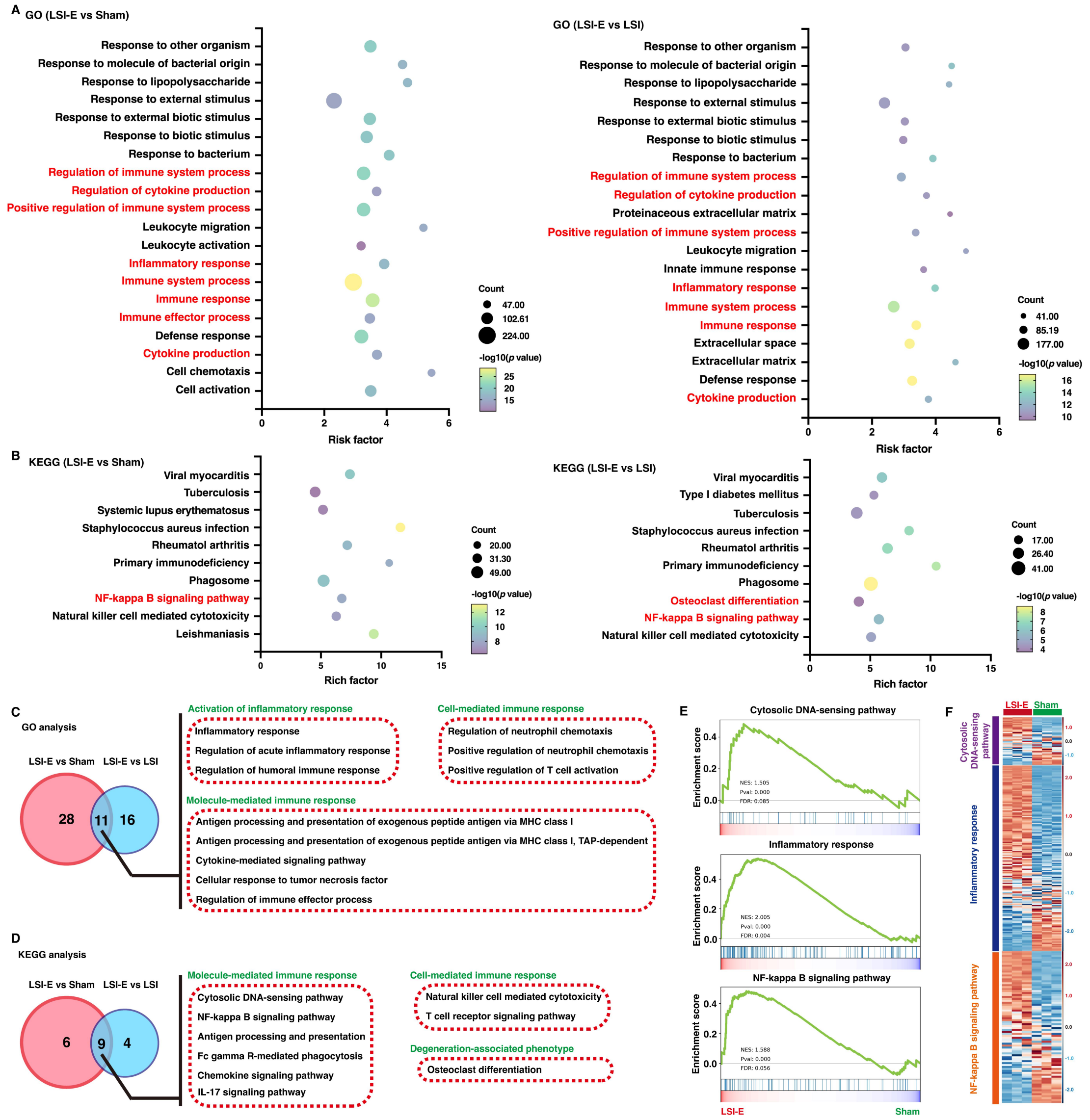

A) Top 20 upregulated GO pathways of DEGs between LSI-E group and Sham group or LSI group. Red-marked pathways were associated with inflammatory response (n = 3 biological independent samples). B) Top 10 upregulated KEGG pathways of DEGs between LSI-E group and Sham group or LSI group. Red-marked pathways were associated with inflammatory response or degenerated phenotypes (n = 3 biological independent samples). C) Overlapping of upregulated inflammatory response-associated GO pathways between LSI-E group and Sham group or LSI group by GSEA. D) Overlapping of upregulated inflammatory response-associated KEGG pathways between LSI-E group and Sham group or LSI group by GSEA. E) Enriched pathways in LSI-E group vs. Sham group by GSEA. F) Gene expression heatmaps of enriched pathways in

LSI-E or Sham group (n = 3 biological independent samples).

Supplementary Fig. 3. Senescent NP cells harbored STING-mediated inflammatory response and disassembly of TRAM1-TREX1 complex.

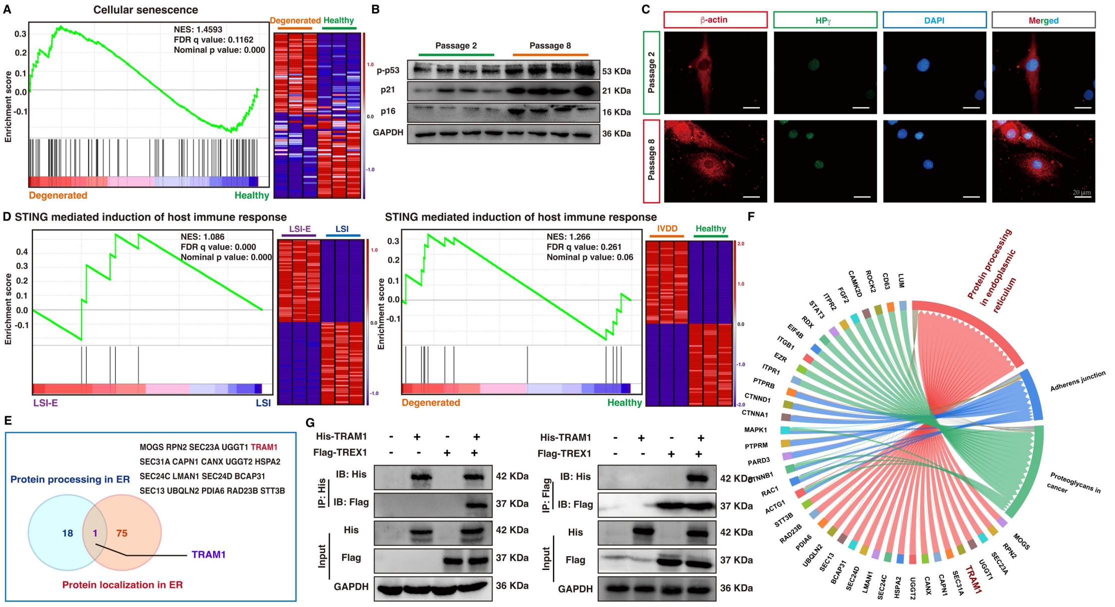

A) Upregulated "Cellular senescence" pathways and expression heatmaps of cellular senescence-associated genes analyzed by GSEA between degenerated and normal NP tissue samples (n = 3 biological independent samples). B) Representative western blotting images of p-p53, p21 and p16 in P2 and P8 NP cells (n = 4 biological independent samples). C) Representative IF staining images of cell morphology via F-actin staining and HP1γ-containing senescence-associated heterochromatin foci (SAHFs) in P8 NP cells, bar: 20 μm (Representative image of three independent experiments). D) Upregulated "STING mediated induction of host immune response" pathways and expression heatmaps of related genes analyzed by GSEA between LSI-E and LSI group and between degenerated and normal NP tissue samples (n = 3 biological independent samples). E) TRAM1 protein was overlapped in two molecular sets involved in "protein processing in ER" (top differential enriched pathway from normal and senescent TREX1 interactome) and "protein localization in ER" (typical GO biological pathway). F) Chordal graph of differential candidate proteins in top 3 enriched KEGG pathways of TREX1 interactome in normal NP cells. G) Exogenous forward and reverse Co-IP assay to detect the interaction of TRAM1 and TREX1 in HEK-293T cells after co-transfected with His-tagged TRAM1 and Flag-tagged TREX1 plasmids (Representative blot of three independent technical experiments).

Supplementary Fig. 4. Loss of TRAM1 disrupted the tethering of TREX1 in the ER, and triggered cGAS-STING axis-mediated NP cell senescence.

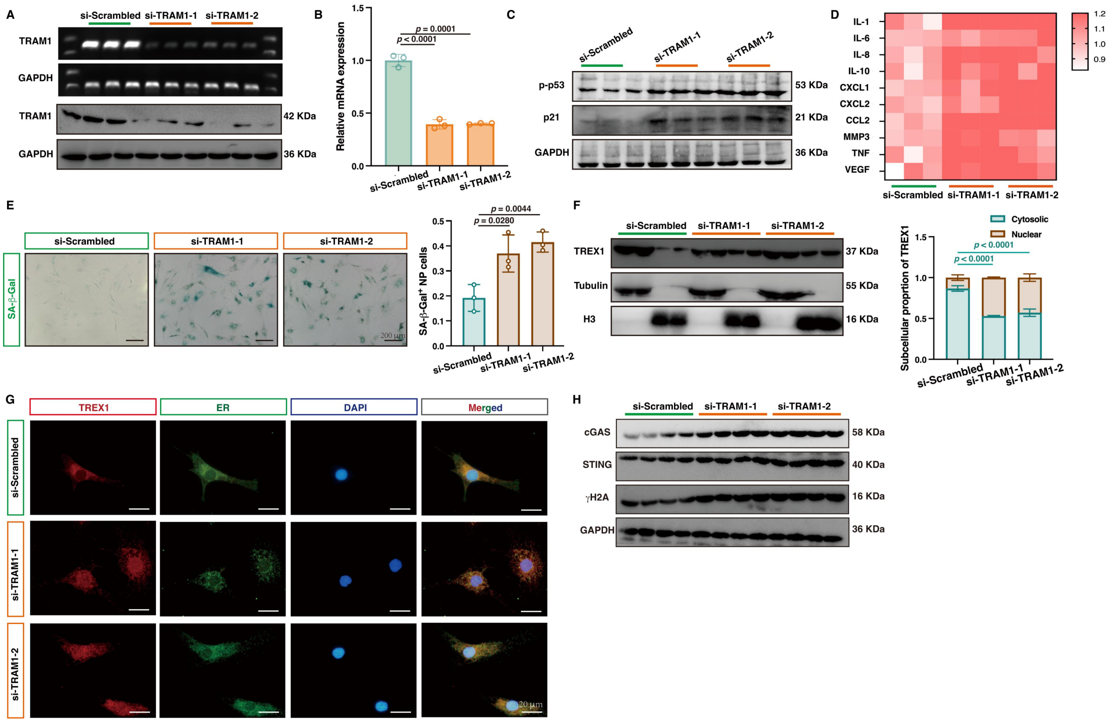

A) Representative agarose gel electrophoresis images of TRAM1 cDNA amplification products via PCR and western blotting images of TREX1 in specific siRNA-induced TRAM1-deficient P2 NP cells (n = 3 biological independent experiments). B) Quantitative analysis to show mRNA expression of TRAM1 genes in specific siRNA-induced TRAM1-deficient P2 NP cells (n = 3 biological independent experiments). C) Representative western blotting images of p-p53 and p21 in siRNA-induced TRAM1-deficient P2 NP cells (n = 3 biological independent experiments). D) Differential expression heatmap of SASP in siRNA-induced TRAM1-deficient P2 NP cells (n = 3 biological independent experiments). E) SA-β-gal staining and quantitative analysis in siRNA-induced TRAM1-deficient P2 NP cells, bar: 20 μm (n = 3 biological independent experiments). F) Representative western blotting images and quantitative analysis of cytosolic and nuclear TREX1 protein in siRNA-induced TRAM1-deficient P2 NP cells (n = 4 biological independent experiments). G) IF staining images of TREX1 in siRNA-induced TRAM1-deficient P2 NP cells, bar: 20 μm (Representative image of three independent experiments). H) Representative western blotting images of cGAS, STING and γH_2A in siRNA-induced TRAM1-deficient P2 NP cells (n = 4 biological independent experiments). A significant *p* value was determined by two-tailed ANOVA (B, E, F). Mean ± SD are shown for (B, E, F).

Supplementary Fig. 5. Upregulation of TRAM1 in senescent NP cells reconstructed TREX1 function and alleviated cGAS-STING axis activation.

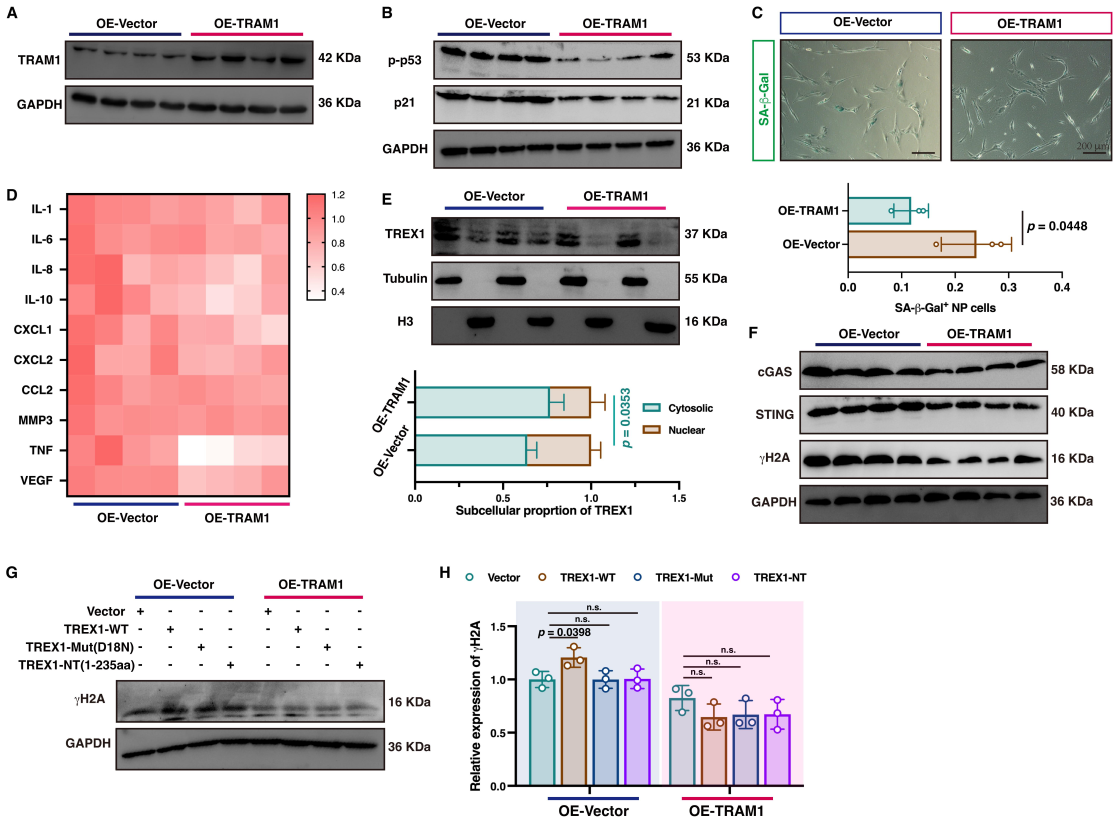

A) Representative western blotting images of TRAM1 in senescent NP cells after transfected with TRAM1-overexpressing plasmid (n = 4 biological independent experiments). B) Representative western blotting images of p-p53 and p21 in TRAM1-overexpressing plasmid transfected P8 NP cells (n = 4 biological independent experiments). C) SA-β-gal staining and quantitative analysis in TRAM1-overexpressing plasmid transfected P8 NP cells (Representative image of three independent experiments). D) Differential expression heatmap of SASP in TRAM1-overexpressing plasmid transfected P8 NP cells, bar: 20 μm (n = 4 biological independent experiments). E) Representative western blotting images and quantitative analysis of cytosolic and nuclear TREX1 protein in TRAM1-overexpressing plasmid transfected P8 NP cells (Representative image of four independent experiments, and quantification of four independent experiments). F) Representative western blotting images of cGAS, STING and γH_2A in TRAM1-overexpressing plasmid transfected P8 NP cells (n = 4 biological independent experiments). G), H) Representative western blotting images and quantitative analysis of γH_2A in TRAM1-overexpressing and different Flag-tagged TREX1 variant plasmids-cotransfected P8 NP

cells (Representative image of four independent technical experiments, and quantification of four independent technical experiments). A significant *p* value was determined by two-tailed unpaired *t* test (E, F) and two-tailed ANOVA (H). Mean ± SD are shown for (E, F, H). n.s. not significant.

Supplementary Fig. 6. TRAM1-loading EXPLOR-engineered EVs alleviated inflammatory senescence phenotype acquisition of NP cells.

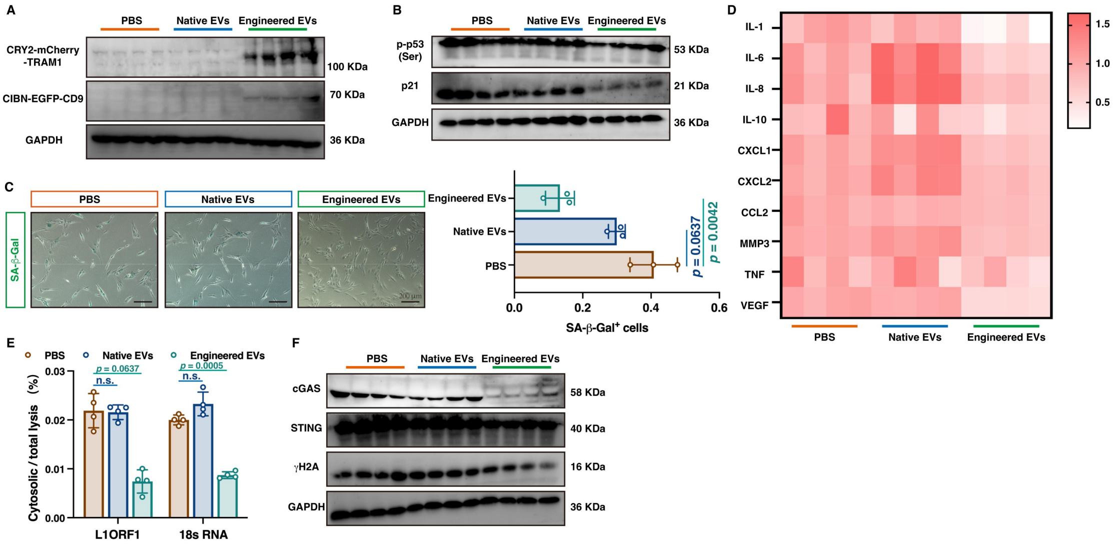

A) Representative western blotting images of CRY2-mCherry-TRAM1 and CIBN-EGFP-CD9 in P8 NP cells after treated with PBS, native or engineered EVs for 72 h (n = 4 biological independent experiments). B) Representative western blotting images of p-p53 and p21 in P8 NP cells after treated with PBS, native or engineered EVs for 72 h (n = 4 biological independent experiments). C) SA-β-gal staining and quantitative analysis in P8 NP cells after treated with PBS, native or engineered EVs for 72 h, bar: 200 μm (Representative image of three independent experiments). D) Differential expression heatmap of SASP in P8 NP cells after treated with PBS, native or engineered EVs for 72 h (n = 4 biological independent experiments). E) Quantitative analysis of western blotting of cytosolic and nuclear TREX1 protein in P8 NP cells after treated with PBS, native or engineered EVs for 72 h (n = 4 biological independent experiments). F) Representative western blotting images of cGAS, STING and γH_2A in P8 NP cells after treated with PBS, native or engineered EVs for 72 h (n = 4 biological independent experiments). A significant *p* value was determined by two-tailed unpaired *t* test (B) and two-tailed ANOVA (E). Mean ± SD are shown for (B, E). n.s. not significant.

Supplementary Fig. 7. Structural design and electrical output performance of TENG.

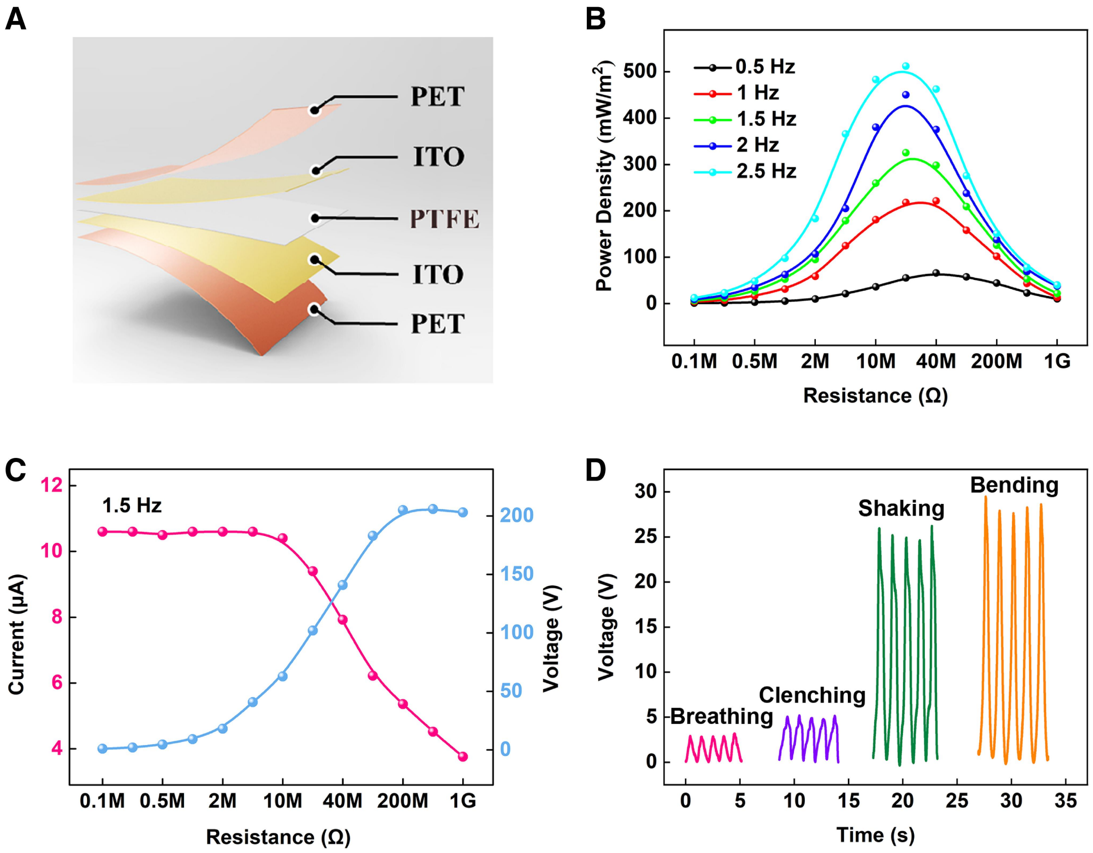

A) Structural schematic of the TENG. B) Relationship between peak power density and external load resistance under various motion frequencies ranging from 0.5 to 2.5 Hz. C) Dependence of the output current and voltage on the external load resistance. D) The open-circuit voltage generated by the TENG in different movements of body. Representative plot of three independent experiments (B-D).

Supplementary Fig. 8. The design and characterization of the triboelectric-responsive MNs with controllable release of EXPLOR-engineered EVs.

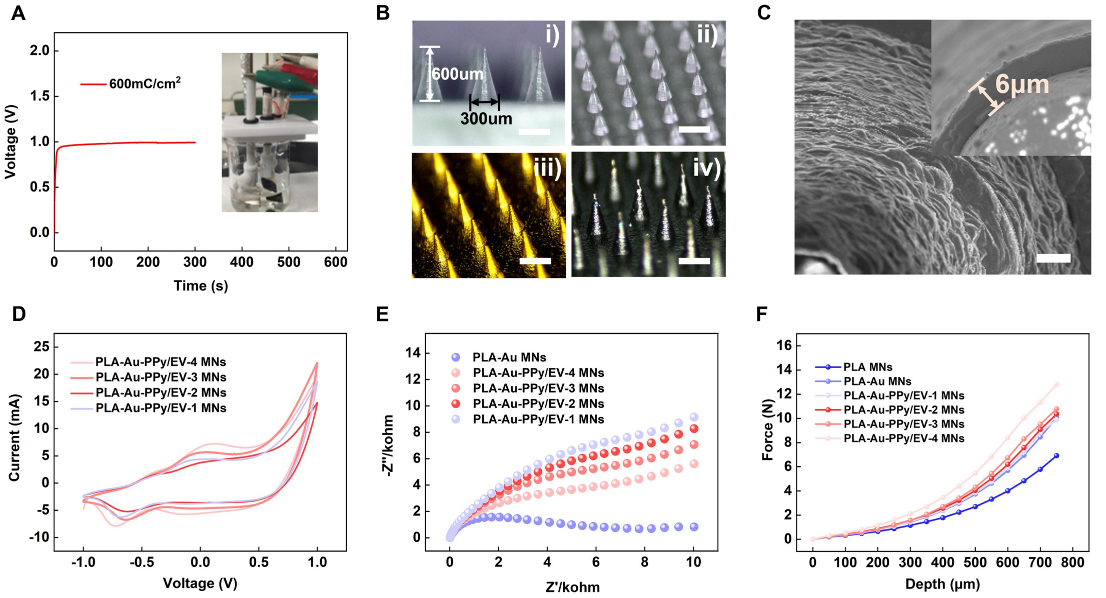

A) The potential of the electropolymerization process of polypyrrole with EXPLOR-engineered EVs. B) The optical pictures of PLA MNs, PLA-Au MNs and PLA-Au-PPy/EV MNs, bar: 300 μm. C) SEM picture of the PLA-Au-PPy/EV MNs, bar: 20 μm. D), E) The CV and EIS curve of PLA MNs, PLA-Au MNs and PLA-Au-PPy/EV MNs. F) The characterization of the ability of MNs to insert into pig skin. Representative plot of three independent experiments (A, D-F).

Supplementary Fig. 9. The experimental procedure of the ability of MNs to insert pig skin.

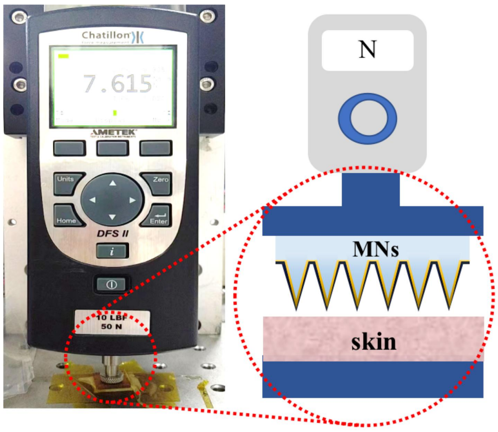

Supplementary Fig. 10. Triboelectric-responsive MNs delivered TRAM1-loading engineered EVs and mitigated inflammatory senescence of NP cells in vitro.

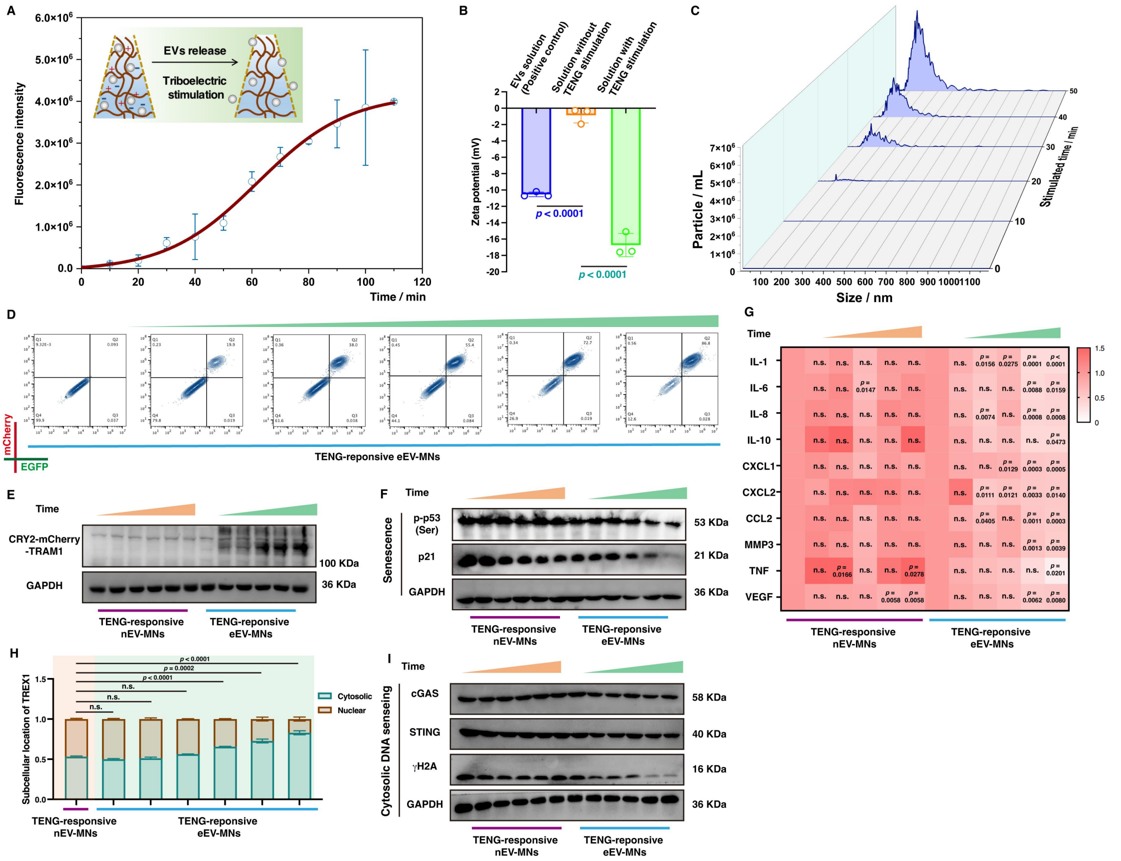

A) EV release dynamics curve via detecting the fluorescence intensity of mCherry-conjugated protein in collected medium at different time points (Representative plot of three independent experiments). B) Zeta-potentials of EVs in collected medium after treatment with triboelectric stimulation (n = 3 biological independent experiments). C) NTA of EVs in collected medium after treatment with different triboelectric stimulation durations. D) Flow cytometry images to analyze the uptake of EVs in P8 NP cells after cultured in the medium with native EV-carrying MNs connected to TENG (triboelectric-responsive nEV-MNs) or TRAM1-engineered EV-carrying MNs connected to TENG (triboelectric-responsive eEV-MNs) with different triboelectric stimulation durations (0, 10, 20, 30, 40, 50 min) (Representative plot of three independent experiments). E) Representative western blotting images of CRY2-mCherry-TRAM1 protein from P8 NP cells after cultured in the medium with EV-carrying MNs connected to TENG with different triboelectric stimulation durations (Representative plot of three independent experiments). F) Representative western blotting images of p-p53 and p21 in P8 NP cells after cultured in the medium with EV-carrying MNs connected to TENG with different triboelectric stimulation

durations (Representative plot of three independent experiments). G) Differential expression heatmap of SASP in P8 NP cells after cultured in the medium with EV-carrying MNs connected to TENG with different triboelectric stimulation durations (Representative plot of four independent experiments). H) Quantitative analysis of western blotting of cytosolic and nuclear TREX1 protein in P8 NP cells after cultured in the medium with EV-carrying MNs connected to TENG with different triboelectric stimulation durations (Quantification of three independent experiments). I) Representative western blotting images of cGAS, STING and γH_2A in P8 NP cells after cultured in the medium with EV-carrying MNs connected to TENG with different triboelectric stimulation durations (Representative plot of three independent experiments). A significant *p* value was determined by two-tailed ANOVA (B, G, H). Mean ± SD are shown for (B, G, H). n.s. not significant.

Supplementary Fig. 11 Illumination-induced TRAM1 loading into EVs was essential for the therapeutic effects of engineered EVs for remodeling TREX1 function and inhibiting NP cell senescent phenotypic acquisition.

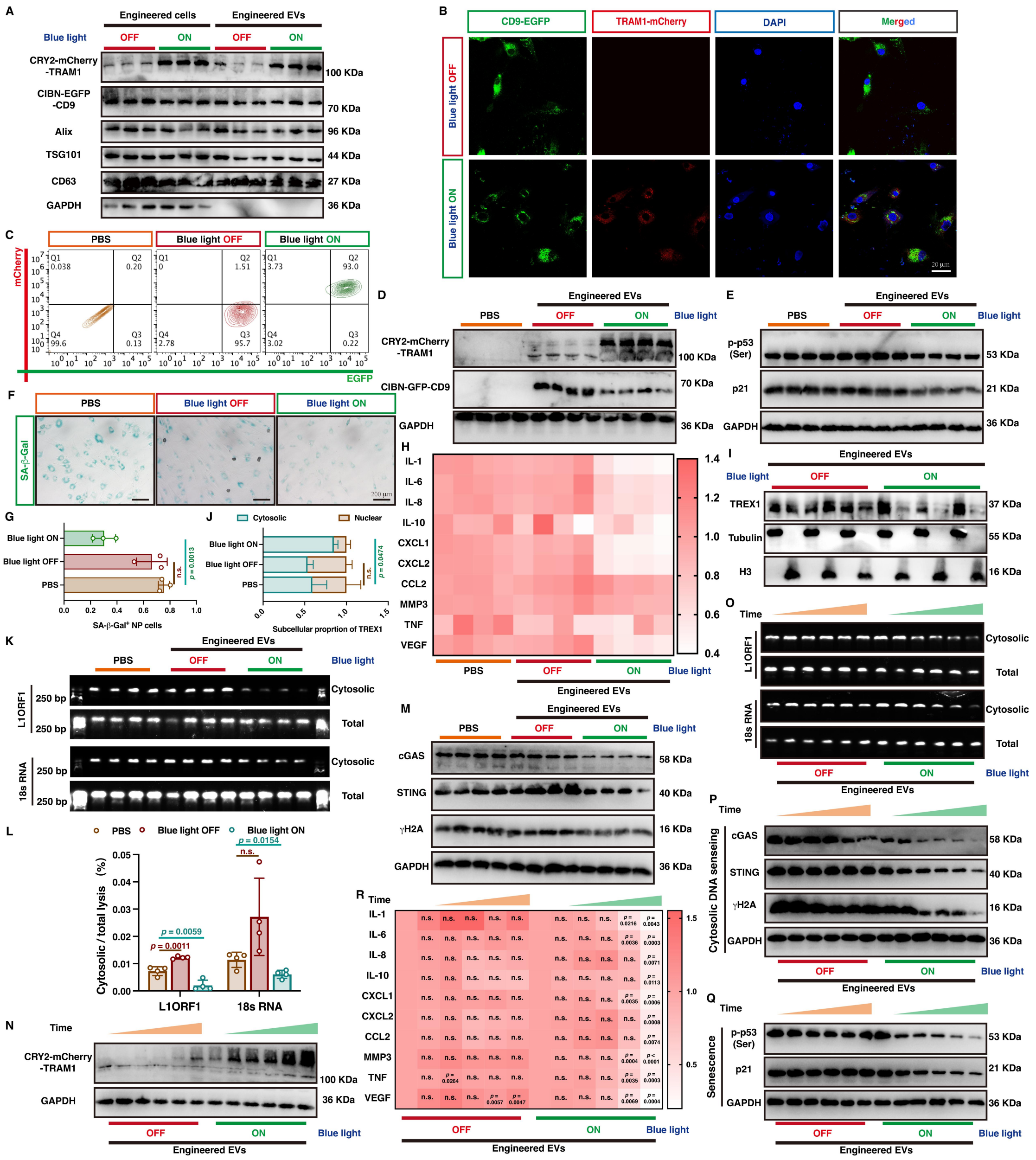

A) Representative western blotting images of CRY2-mCherry-TRAM1, CIBN-EGFP-CD9 and EV markers in HEK-293T cells and isolated engineered EVs from HEK-293T cells after cotransfection with CIBN-EGFP-CD9 and CRY2-mCherry-TRAM1 plasmids with ("Blue light ON") or without ("Blue light OFF") 460-nm laser stimulation (20 μW/cm^2) (n = 3 biological independent experiments). B) Representative IF staining images of EGFP and mCherry in P8 NP cells after treatment with engineered EVs for 72 h, bar: 20 μm (Representative image of three independent experiments). C) Flow cytometry images to analyze the uptake of EVs in P8 NP cells

after treatment with engineered EVs for 72 h (Representative blot of three independent experiments). D) Representative western blotting images of CRY2-mCherry-TRAM1, CIBN-EGFP-CD9 from P8 NP cells after treated with PBS or engineered EVs for 72 h (n = 4 biological independent experiments). E) Representative western blotting images of p-p53 and p21 in P8 NP cells after treated with PBS or engineered EVs for 72 h (n = 4 biological independent experiments). F) SA-β-gal staining and G) quantitative analysis of SA-β-gal$^+$ NP cell proportion in P8 NP cells after treated with PBS, native or engineered EVs for 72 h, bar: 200 μm (Representative blot of three independent experiments, and quantification of three independent experiments). H) Differential expression heatmap of SASP in P8 NP cells after treated with PBS or engineered EVs for 72 h (n = 4 biological independent experiments). I) Representative western blotting and J) quantitative analysis of cytosolic and nuclear TREX1 protein in P8 NP cells after treated with PBS or engineered EVs for 72 h (Representative blot of three independent experiments, and quantification of three independent experiments). K) Representative agarose gel electrophoresis images and L) quantitative analysis of DNA fragments from the cytosolic component in P8 NP cells after treatment with PBS or engineered EVs for 72 h (Representative blot of four independent experiments, and quantification of four independent experiments). M) Representative western blotting images of cGAS, STING and γH_2A in P8 NP cells after treated with PBS or engineered EVs for 72 h (n = 4 biological independent experiments). N) Representative western blotting images of CRY2-mCherry-TRAM1 protein from P8 NP cells after cultured in the medium with engineered EV-carrying MNs connected to TENG with different triboelectric stimulation durations (Representative blot of three independent experiments). O) Representative electrophoresis images of DNA fragments from the cytosolic component in P8 NP cells after culture in medium with engineered EV-carrying MNs connected to TENGs with different triboelectric stimulation durations (Representative blot of three independent experiments). P) Representative western blotting images of cGAS, STING and γH_2A in P8 NP cells after cultured in the medium with engineered EV-carrying MNs connected to TENG with different triboelectric stimulation durations (Representative blot of three independent experiments). Q) Representative western blotting images of p-p53 and p21 in P8 NP cells after cultured in the medium with engineered EV-carrying MNs connected to TENG with different triboelectric stimulation durations (Representative blot of three independent experiments). R) Differential

expression heatmap of SASP in P8 NP cells after cultured in the medium with engineered EV-carrying MNs connected to TENG with different triboelectric stimulation durations (n = 3 biological independent experiments). A significant p value was determined by two-tailed ANOVA (G, J, L, R). Mean ± SD are shown for (G, J, L, R). n.s. not significant.

Supplementary Fig. 12 EV-carrying MN systems didn’t contribute to unexpected inflammatory response in the inserted skin and paravertebral tissues.

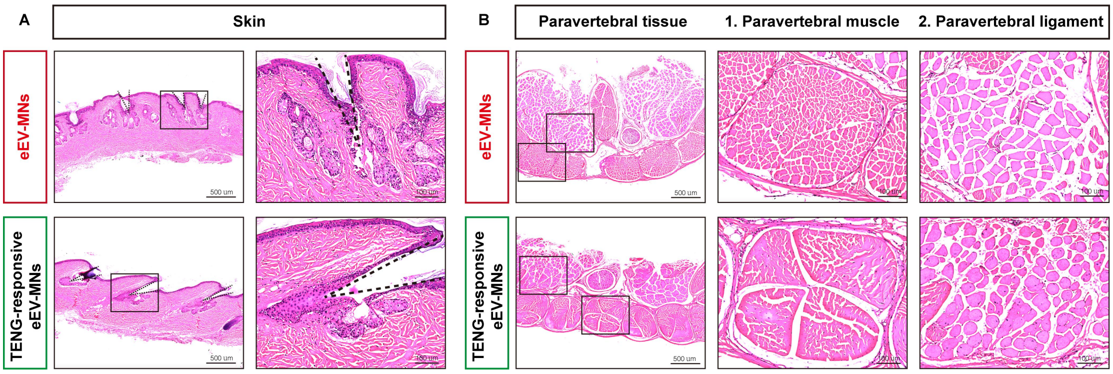

A) Representative images of the H&E staining of skin and subcutaneous tissues from different groups (eEV-MNs and TENG-responsive eEV-MNs). Scale bars, 500 μm, 100 μm. B) Representative images of the H&E staining of paravertebral tissues including paravertebral muscles and paravertebral ligaments from different groups (eEV-MNs and TENG-responsive eEV-MNs). Scale bars, 500 μm, 100 μm. Representative images of five independent experimental replicates (A, B).

Supplementary Fig. 13 Self-powered triboelectric-responsive MN system alleviated the senescence and inflammatory response of NP cells in rat coccygeal IVDD.

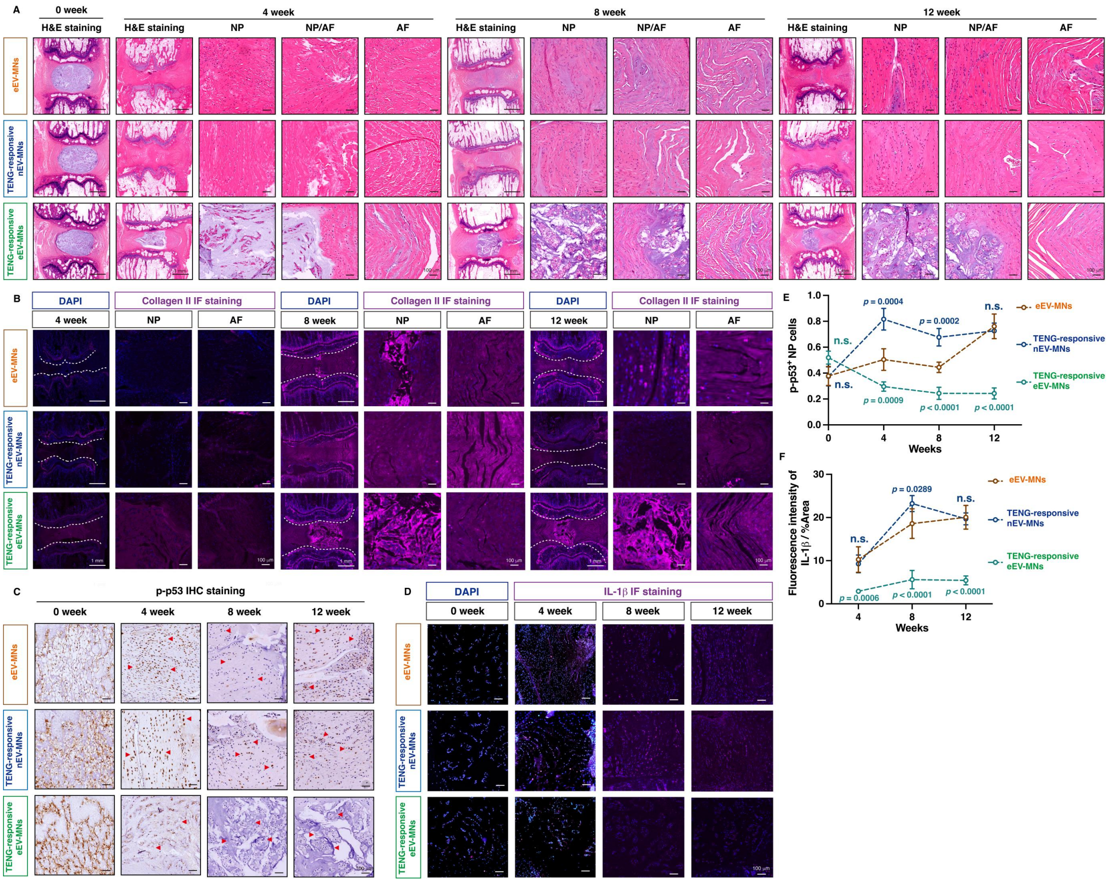

A) H&E staining of C7-C8 IVDs from rats with the indicated treatments for 4, 8, 12 weeks. B) IF staining of type II collagen in C7-C8 IVDs from rats with the indicated treatments for 4, 8 and 12 weeks. C) IHC staining of p-p53 in C7-C8 IVDs from rats with the indicated treatments for 4, 8 and 12 weeks. The arrows indicated p-p53$^+$ NP cells. D) IF staining of IL-1β in C7-C8 IVDs from rats with the indicated treatments for 4, 8 and 12 weeks. E) Quantitative analysis of the p-p53$^+$ NP cell proportion in C7-C8 IVDs from rats with the indicated treatments for 4, 8 and 12 weeks. F) Quantitative analysis of the fluorescence intensity of IL-1β in C7-C8 IVDs from rats with the indicated treatments for 4, 8 and 12 weeks. Representative images of five independent experimental replicates (A-D), and quantification of five independent biological replicates (E, F). A significant p value was determined by two-tailed ANOVA (E, F). Mean ± SD are shown for (E, F). n.s. not significant.

Supplementary Fig. 14 Illumination-induced TRAM1 loading into EVs was essential for the therapeutic effects of engineered-EV carrying MN system for alleviating needle puncture-induced rat coccygeal IVDD.

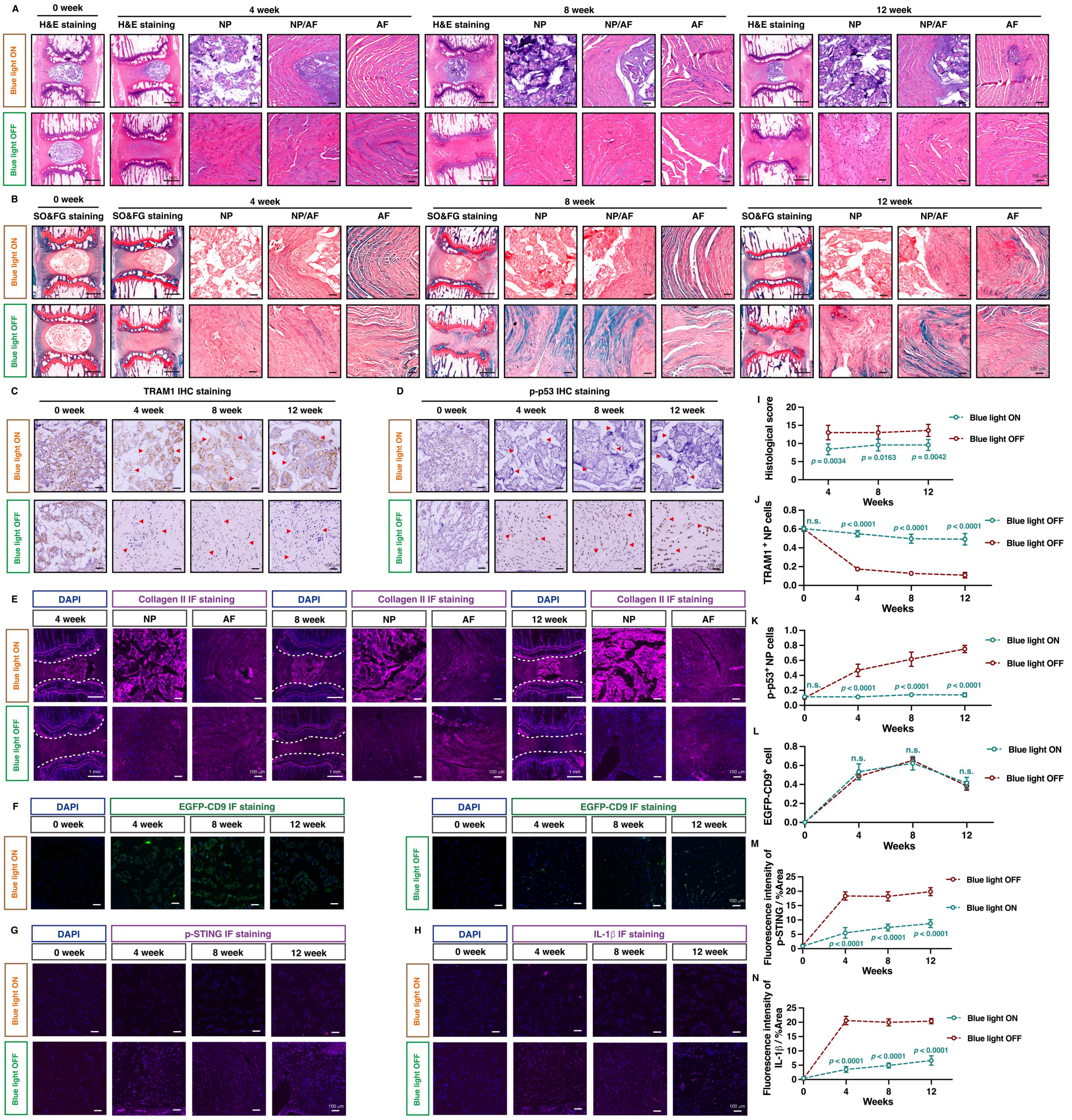

Isolated engineered EVs from HEK-293T cells after cotransfection with CIBN-EGFP-CD9 and CRY2-mCherry-TRAM1 plasmids with ("Blue light ON") or without ("Blue light OFF") 460-nm laser stimulation (20 μW/cm^2) were loaded into triboelectric-responsive MN system. And rat coccygeal IVDs for needle puncture model wearing different equipments (TENG-responsive "Blue light ON" EV-MNs or TENG-responsive "Blue light OFF" EV-MNs) were performed (n = 5 rats per group). A) H&E staining of C7-C8 IVDs from rats with the indicated treatments for 4, 8, 12 weeks. B) SO&FG staining of C7-C8 IVDs from rats with the indicated treatments for 4, 8 and 12 weeks. C) IHC staining of TRAM1 in C7-C8 IVDs from rats with the indicated treatments for

4, 8 and 12 weeks. D) IHC staining of p-p53 in C7-C8 IVDs from rats with the indicated treatments for 4, 8 and 12 weeks. E) IF staining of type II collagen in C7-C8 IVDs from rats with the indicated treatments for 4, 8 and 12 weeks. F) IF staining of EGFP-CD9 in C7-C8 IVDs from rats with the indicated treatments for 4, 8, 12 weeks. G) IF staining of p-STING in C7-C8 IVDs from rats with the indicated treatments for 4, 8 and 12 weeks. H) IF staining of IL-1β in C7-C8 IVDs from rats with the indicated treatments for 4, 8 and 12 weeks. I) Histological score of C7-C8 IVDs from rats with the indicated treatments for 4, 8, and 12 weeks. J) Quantitative analysis of the $TRAM1^+$ NP cell proportion in C7-C8 IVDs from rats with the indicated treatments for 4, 8 and 12 weeks. K) Quantitative analysis of the p-$p53^+$ NP cell proportion in C7-C8 IVDs from rats with the indicated treatments for 4, 8 and 12 weeks. L) Quantitative analysis of EGFP-$CD9^+$ NP cell proportions in C7-C8 IVDs from rats with the indicated treatments for 4, 8 and 12 weeks. M) Quantitative analysis of the fluorescence intensity of p-STING in C7-C8 IVDs from rats with the indicated treatments for 4, 8 and 12 weeks. N) Quantitative analysis of the fluorescence intensity of IL-1β in C7-C8 IVDs from rats with the indicated treatments for 4, 8 and 12 weeks. Representative images of five independent experimental replicates (A-H), and quantification of five independent biological replicates (I-N). A significant *p* value was determined by two-tailed unpaired *t* test (I-N). Mean ± SD are shown for (I-N). n.s. not significant.

Supplementary Fig. 15 The therapeutic efficacy of different delivery strategies for alleviating rat coccygeal IVDD.

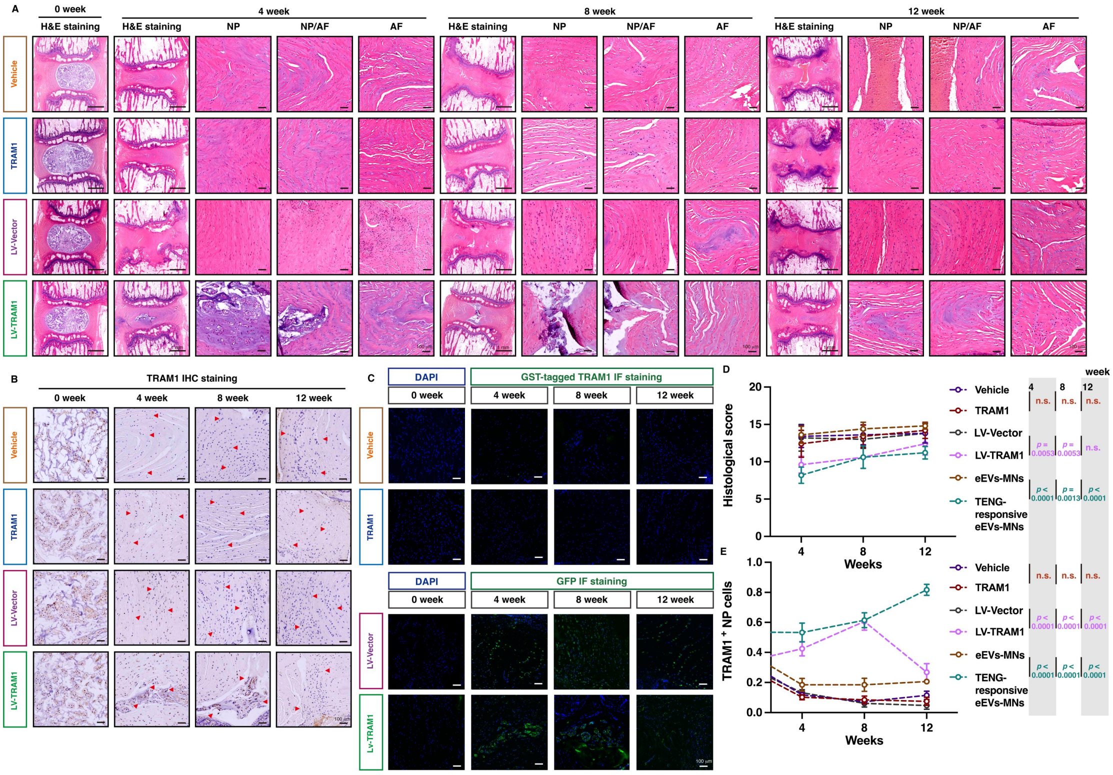

To compare the delivery efficacy of different delivery strategies, we used exogenous GST-tagged TRAM1 protein solution or TRAM1-overpressing plasmid-carrying LVs to inject into the NP tissues of needle puncture-induced rat coccygeal IVDD model (n = 5 rats per group). A) H&E staining of C7-C8 IVDs from rats with the indicated treatments for 4, 8, 12 weeks. B) IHC staining of TRAM1 in C7-C8 IVDs from rats with the indicated treatments for 4, 8 and 12 weeks. C) IF staining of GST or GFP in C7-C8 IVDs from rats with the indicated treatments for 4, 8, 12 weeks. D) Histological score of C7-C8 IVDs from rats with the indicated treatments for 4, 8, and 12 weeks. E) Quantitative analysis of the $TRAM1^{+}$ NP cell proportion in C7-C8 IVDs from rats with the indicated treatments for 4, 8 and 12 weeks). Representative images of five independent experimental replicates (A-C), and quantification of five independent biological replicates (D, E). A significant *p* value was determined by two-tailed unpaired *t* test (D, E). Mean ± SD are shown for (D, E). n.s. not significant.

Supplementary Table 1. Characteristics details of the volunteers used for RNA-sequencing

Case No.	Age (Years)	Gender	Diagnosis	Disc level	Pfirrmann MRI grading
Case 1	15	Male	Idiopathic scoliosis	T12-L1	I
Case 2	14	Female	Idiopathic scoliosis	L1-L2	I
Case 3	17	Male	Idiopathic scoliosis	L1-L2	I
Case 4	35	Male	Lumbar disc herniation	L4-L5	IV
Case 5	43	Female	Lumbar disc herniation	L5-S1	IV
Case 6	56	Male	Lumbar disc herniation	L5-S1	IV

Supplementary Table 2. Primer sequences used in PCR genotyping

Gene	Forward (5'-3')	Reverse (5'-3')
Homo IL-1β	GGTTGAGTTTAAGCCAATCCA	TGCTGACCTAGGCTTGATGA
Homo IL-6	GCCCAGCTATGAACTCCTTCT	GAAGGCAGCAGGCAACAC
Homo IL-8	AGACAGCAGAGCACACAAGC	ATGGTTCCTTCCGGTGGT
Homo IL-10	TGCCTTCAGCAGAGTGAAGA	GCTTGGCAACCCAGGTAA
Homo CXCL1	GCTGAACAGTGACAAATCCAAC	CTTCAGGAACAGCCACCAGT
HomoCXCL2	CCCATGGTTAAGAAAATCATCG	CTTCAGGAACAGCCACCAAT
Homo CCL2	AGTCTCTGCCGCCCTTCT	GTGACTGGGGCATTGATTG
Homo TNF-α	CAGCCTCTTCTCCTTCCTGAT	GCCAGAGGGCTGATTAGAGA
Homo VEGF	AGGGCAGAATCATCACGAAGT	AGGGTCTCGATTGGATGGCA
Homo MMP3	CAAAACATATTTCTTTGTAGAGGACAA	TTCAGCTATTTGCTTGGGAAA
Homo TRAM1	ATTAACAGGCGAATGCACTTCT	CCCTCCATAAGATAGTTGGGTCT
Homo GAPDH	CAAGAAGGTGAAGCAGG	TCAAAGGTGGAGGAGTGGGT
Homo g-L1ORF1	AGAACGCCACAAAGATACTCCTCG	CTCTCTTCTGGCTTGTAGGGTTTCTG
Homo g-RNA18S	GTAACCCGTTGAACCCCATT	CCATCCAATCGGTAGTAGCG

Supplementary Table 3. siRNA sequences used in siRNA transfection

Sequence name	Sense (5'-3')	Antisense (5'-3')
Homo si-Scrambled	UUCUCCGAACGUGUCACGUTT	ACGUGACACGUUCGGAGAATT
Homo si-TRAM1-1	GAUAAUUAUUCAUGCCGUATT	UACGGCAUGAAUAAUUAUCTT
Homo si-TRAM2-2	AGGCAUUUAUGAUGUGGAATT	UUCCACAUCAUAAAUGCCUTT

Supplementary Table 4. Antibody information

Antibody	Company	Catalog#	Application/ Dilution
anti-p-p53 (Ser15)	Abcam	ab278683	IHC (1:150); WB (1: 500)
anti-p21 (CDKN1A)	Cell Signaling Technology	2947	WB (1: 500)
anti-γH_2A	Abcam	ab81299	WB (1: 500)
anti-p16INK4a	Affinity	AF5484	WB (1: 500)
anti-HP1γ	Abcam	ab213167	IF (1:250)
anti-cGAS	Cell Signaling Technology	83623	WB (1: 500)
anti-STING	Cell Signaling Technology	13647	WB (1: 1000)
anti-pSTING	Cell Signaling Technology	50907	IHC (1: 500)
anti-TREX1	Cell Signaling Technology	#15107	IP (4 µg/1000 µg); WB (1: 500)
anti-TRAM1	Abcam	ab96106	IP (6 µg/1000 µg); WB (1: 500); IHC (1: 100)
anti-mCherry	Proteintech	26765-1-AP	WB (1: 1000)
anti-EGFP	Proteintech	66002-1-Ig	WB (1: 2000)
anti-Alix	Abcam	ab275377	WB (1: 1000)
anti-CD63	Abcam	ab134045	WB (1: 1000)
anti-TSG101	Abcam	ab125011	WB (1: 1000)
anti-collagen II	Proteintech	28459-1-AP	IF (1:100)
anti-IL-1β	Abcam	ab254360	IF (1:100)
anti-Flag tag epitope	Proteintech	66008-4-AP	IP (5 µg/1000 µg); WB (1: 500)
anti-His tag epitope	Proteintech	66005-1-Ig	IP (5 µg/1000 µg); WB (1: 500)
anti-GAPDH	Proteintech	60004-1-Ig	WB (1: 2000)
anti-β-actin	Proteintech	66009-1-Ig	WB (1: 2000)
anti-β-Tubulin	Proteintech	66240-1-Ig	WB (1: 2000)
anti-H3	Proteintech	17168-1-AP	WB (1: 2000)
