# Supplementary material for: Self-powered triboelectric-responsive microneedles with controllable release of optogenetically engineered extracellular vesicles for intervertebral disc degeneration repair
Source: Nat Commun. 2024 Jul 9;15:5736. doi: 10.1038/s41467-024-50045-1 (PMC11233569; doi:10.1038/s41467-024-50045-1)
Supplement: Supplementary file 3 — Description Of Additional Supplementary File [file 41467_2024_50045_MOESM3_ESM.pdf]

Description of addition supplementary file

Supplementary Mov. 1 : Rat wearing TENG-responsive MNs with controllable release of EXPLOR-EVs for IVDD repair.
