# Supplementary material for: Self-powered triboelectric-responsive microneedles with controllable release of optogenetically engineered extracellular vesicles for intervertebral disc degeneration repair
Source: Nat Commun. 2024 Jul 9;15:5736. doi: 10.1038/s41467-024-50045-1 (PMC11233569; doi:10.1038/s41467-024-50045-1)
Supplement: Supplementary file 5 — Reporting summary [file 41467_2024_50045_MOESM5_ESM.pdf]

Corresponding author(s): Cao Yang; Zhen Wen; Xuhui Sun; Bingjin Wang

Last updated by author(s): May 25, 2024

Reporting Summary

Nature Portfolio wishes to improve the reproducibility of the work that we publish. This form provides structure for consistency and transparency in reporting. For further information on Nature Portfolio policies, see our Editorial Policies and the Editorial Policy Checklist.

Statistics

For all statistical analyses, confirm that the following items are present in the figure legend, table legend, main text, or Methods section.

n/a	Confirmed	
☐	☒	The exact sample size (*n*) for each experimental group/condition, given as a discrete number and unit of measurement
☐	☒	A statement on whether measurements were taken from distinct samples or whether the same sample was measured repeatedly
☐	☒	The statistical test(s) used AND whether they are one- or two-sided<br>*Only common tests should be described solely by name; describe more complex techniques in the Methods section.*
☒	☐	A description of all covariates tested
☐	☒	A description of any assumptions or corrections, such as tests of normality and adjustment for multiple comparisons
☐	☒	A full description of the statistical parameters including central tendency (e.g. means) or other basic estimates (e.g. regression coefficient) AND variation (e.g. standard deviation) or associated estimates of uncertainty (e.g. confidence intervals)
☐	☒	For null hypothesis testing, the test statistic (e.g. *F*, *t*, *r*) with confidence intervals, effect sizes, degrees of freedom and *P* value noted<br>*Give P values as exact values whenever suitable.*
☒	☐	For Bayesian analysis, information on the choice of priors and Markov chain Monte Carlo settings
☒	☐	For hierarchical and complex designs, identification of the appropriate level for tests and full reporting of outcomes
☒	☐	Estimates of effect sizes (e.g. Cohen's *d*, Pearson's *r*), indicating how they were calculated

Our web collection on statistics for biologists contains articles on many of the points above.

Software and code

Policy information about availability of computer code

Data collection	Bio-rad CFX Connect Real-Time System was used for PCR; ChemiDoc MP Imaging System (Bio-Rad, 12003154 Hercules, CA, USA) was used for Western blotting; Transmission electron microscopy (H-7000FA, Hitachi LTD, Japan) was used to show the morphology of extracellular vesicles; NANOSIGHT NS300 system (Malvern, UK) was used to analyze the size and concentration of extracellular vesicles; Zeta potential analysis (Malvern Zetasizer Nano ZS90, UK) was used to detect the stability of extracellular vesicles; Then, FACSCalibur flow cytometer (BD Biosciences, USA) was used to perform flow cytometer; Orbitrap Fusion Lumos (Thermo Scientific, USA) was applied for mass spectrometric detection.
Data analysis	Image J (V1.52v); Graphpad Prism 9.3.0; Stats R package (3.6.1); DESeq2 R-package; Gene Set Enrichment Analysis (GSEA) v4.2.3; Proteome Discoverer (V1.4)

For manuscripts utilizing custom algorithms or software that are central to the research but not yet described in published literature, software must be made available to editors and reviewers. We strongly encourage code deposition in a community repository (e.g. GitHub). See the Nature Portfolio guidelines for submitting code & software for further information.

Data

Policy information about availability of data

All manuscripts must include a data availability statement. This statement should provide the following information, where applicable:

- Accession codes, unique identifiers, or web links for publicly available datasets
- A description of any restrictions on data availability
- For clinical datasets or third party data, please ensure that the statement adheres to our policy

The RNA-sequencing data generated in this study have been deposited in the Gene Expression Omnibus (GEO) database under accession code GSE266883. The interacting protein candidates binding with TREX1 are available in Source Data files. The information of the volunteers enrolled in the study, primer sequences used in PCR genotyping, siRNA sequences used in siRNA transfection and antibodies used in this study are provided in the Supplementary Information (Supplementary Table 1, 2, 3 and 4). All data supporting the findings of this study are available within the article and its supplementary files. Any additional requests for information can be directed to, and will be fulfilled by, the corresponding authors. Source data are provided with this paper.

Research involving human participants, their data, or biological material

Policy information about studies with human participants or human data. See also policy information about sex, gender (identity/presentation), and sexual orientation and race, ethnicity and racism.

Reporting on sex and gender	Sex and gender were not considered in our study design, but there was no sex or gender difference involving human participants in our study.
Reporting on race, ethnicity, or other socially relevant groupings	Race, ethnicity or other socially relevant groupings were not considered in our study design.
Population characteristics	The NP tissues samples were obtained from patients who had idiopathic scoliosis or lumbar intervertebral disc herniation and underwent orthopedic surgery involving in lumbar segments. For non-degenerated group, there were 2 male patients and 1 female patients with age ranges from 14 to 17 years old. For the degenerated group, there also were 2 male patients and 1 female patients with age ranges from 35 to 56 years old.
Recruitment	Healthy human NP tissue samples were collected from patients who had idiopathic scoliosis and underwent orthopedic surgery involving in lumbar segments, and the MRI-T2 weighed image-based Pfirrmann grading system identified the IVD tissue as Grade I. Degenerated NP tissue samples were collected from patients with lumbar intervertebral disc herniation who were undergoing discectomy, and the Pfirrmann grading system evaluated the IVD tissue as Grade III to Grade IV. Furthermore, some patients without cardio-cerebro-vascular disease, cancers, infection, immune and endocrine diseases or organ dysfunction and approved the application of NP tissue samples in scientific studies. Three pairs, including three non-degenerated and degenerated samples, were used for transcriptome RNA sequencing. However, unavoidable operational errors at the time of sampling may cause results to be inconsistent with the actual degree of disease in patients, thus affecting the results of the study and slightly biased from the actual situation.
Ethics oversight	Patient medical data consultation, NP tissue sample collection and application, animal obtainment, animal surgical operation and animal sample collection, were approved by Ethics Committee of Tongji Medical College, Huazhong University of Science and Technology (No. S341).

Note that full information on the approval of the study protocol must also be provided in the manuscript.

Field-specific reporting

Please select the one below that is the best fit for your research. If you are not sure, read the appropriate sections before making your selection.

☒ Life sciences ☐ Behavioural & social sciences ☐ Ecological, evolutionary & environmental sciences

For a reference copy of the document with all sections, see nature.com/documents/nr-reporting-summary-flat.pdf

Life sciences study design

All studies must disclose on these points even when the disclosure is negative.

Sample size	No statistical methods were used to predetermine sample size. The sample sizes were determined based on previous studies with similar experiments (Nature Communications vol. 9,1 5051. 28 Nov. 2018; Nature Communications vol. 12, 1 5213. 3 Sep. 2021; Nature Communications 13,1 1469. 18 Mar. 2022). For all the experiments, at least three separate technical experiments or at least triplicate biological samples were performed, which allowed sufficient statistics to perform unpaired student t' test or ANOVA analysis, and gave p values to indicate the significance.
Data exclusions	No data was excluded.

Replication	All the experiments were replicated. In general, at least three separate technical experiments or at least triplicate biological samples were performed determined based on the experiments.
Randomization	All samples were randomly assigned, and analyzed together in each experiment.
Blinding	The investigators were blinded to group allocation during data collection and analysis.

Reporting for specific materials, systems and methods

We require information from authors about some types of materials, experimental systems and methods used in many studies. Here, indicate whether each material, system or method listed is relevant to your study. If you are not sure if a list item applies to your research, read the appropriate section before selecting a response.

Materials & experimental systems

n/a	Involved in the study
☐	☒ Antibodies
☐	☒ Eukaryotic cell lines
☒	☐ Palaeontology and archaeology
☐	☒ Animals and other organisms
☒	☐ Clinical data
☒	☐ Dual use research of concern
☒	☐ Plants

Methods

n/a	Involved in the study
☒	☐ ChIP-seq
☐	☒ Flow cytometry
☒	☐ MRI-based neuroimaging

Antibodies

Antibodies used

The information of all the antibodies used in the study were listed in the Supplementary Table 4.

Validation

The validation information of all the antibodies could be searched in the manufacturer's websites according to the source and identifier:
Human anti-phosphorylated p53 (Ser15), IHC (1:150)/WB (1:500), Abcam, ab278683, https://www.abcam.cn/products/primary-antibodies/p53-phospho-s15-antibody-p53s15-1c11-ab278683.html;
Human anti-p21 (CDKN1A), WB (1:500), CST, 2947, https://www.cellsignal.cn/products/primary-antibodies/p21-waf1-cip1-12d1-rabbit-mab/2947?site-search-type=Products&N=4294956287&Ntt=2947&fromPage=plp&_requestid=3118145;
Human gammar-H2A, WB (1:500), Abcam, ab81299, https://www.abcam.cn/products/primary-antibodies/gamma-h2ax-phospho-s139-antibody-ep8542y-ab81299.html;
Human anti-p16, WB (1:500), Affinity, AF5484, https://www.affbiotech.cn/goods-4789-AF5484-CDKN2A_p16INK4a_Antibody.html;
Human anti-HP1-gammar, IF, Abcam, ab213167, https://www.abcam.cn/products/primary-antibodies/hp1-gammacbx3-antibody-epr19803-ab213167.html;
Human anti-cGAS, WB (1:500), CST, 83623, https://www.cellsignal.cn/products/primary-antibodies/cgas-e9g9g-rabbit-mab/83623?site-search-type=Products&N=4294956287&Ntt=83623&fromPage=plp&_requestid=3118440;
Human anti-STING, WB (1:1000), CST, 13647, https://www.cellsignal.cn/products/primary-antibodies/sting-d2p2f-rabbit-mab/13647?site-search-type=Products&N=4294956287&Ntt=13647&fromPage=plp&_requestid=3118529;
Human anti-phosphorylated STING, IHC (1:500), CST, 50907, https://www.cellsignal.cn/products/primary-antibodies/phospho-sting-ser366-e9a9k-rabbit-mab/50907?site-search-type=Products&N=4294956287&Ntt=50907&fromPage=plp&_requestid=3118578;
Human anti-TREX1, IP (1: 250)/WB (1:500), CST, 15107, https://www.cellsignal.cn/products/primary-antibodies/trex1-d8e2o-rabbit-mab/15107?site-search-type=Products&N=4294956287&Ntt=15107&fromPage=plp&_requestid=3118674;
Human anti-TRAM1, IP (1:250)/WB (1:500), Abcam, ab96106, https://www.abcam.cn/products/primary-antibodies/tram1tram-antibody-ab96106.html;
Human anti-mCherry, WB (1: 1000), Proteintech, 26765-1-AP, https://www.ptgcn.com/products/mCherry-Antibody-26765-1-AP.htm;
Human anti-GFP, WB (1: 1000), Proteintech, 66002-1-Ig, https://www.ptgcn.com/products/eGFP-Antibody-66002-1-Ig.htm;
Human anti-Alix, WB (1: 1000), Abcam, ab275377, https://www.abcam.cn/products/primary-antibodies/alix-antibody-epr23653-32-ab275377.html;
Human anti-CD63, WB (1:1000), Abcam, ab134045, https://www.abcam.cn/products/primary-antibodies/cd63-antibody-epr5702-late-endosome-marker-ab134045.html;
Human anti-TSG101, WB (1:1000), Abcam, ab125011, https://www.abcam.cn/products/primary-antibodies/tsg101-antibody-epr7130b-ab125011.html;
Rat anti-collagen II, IF (1:100), Proteintech, 28459-1-AP, https://www.ptgcn.com/products/Collagen-Type-II-Antibody-28459-1-AP.htm#product-information;
Rat anti-IL-1, IF (1:100), Abcam, ab254360, https://www.abcam.cn/products/primary-antibodies/il-1-beta-antibody-epr23851-127-ab254360.html;
Human anti-Flag, IP (1:200)/WB (1: 500), Proteintech, 66008-4-AP, https://www.ptgcn.com/products/Flag-tag-Antibody-66008-4-Ig.htm;
Human anti-His, IP (1:200)/WB (1:500), Proteintech, 66005-1-Ig, https://www.ptgcn.com/products/His-Tag-Antibody-66005-1-Ig.htm;
Human anti-GAPDH (1:2000), WB, Proteintech, 60004-1-Ig, https://www.ptgcn.com/products/GAPDH-Antibody-60004-1-Ig.htm;
Human anti-beta-actin, WB (1:2000), Proteintech, 66009-1-Ig, https://www.ptgcn.com/products/Pan-Actin-Antibody-66009-1-Ig.htm;
Human anti-H3, WB (1:2000), Proteintech, 17168-1-AP, https://www.ptgcn.com/products/Histone-H3-Antibody-17168-1-AP.htm.

Eukaryotic cell lines

Policy information about cell lines and Sex and Gender in Research

Cell line source(s)	Human embryonic kidney 293T cell line was purchased from the American Type Tissue Culture Collection.
Authentication	We performed cell line identification by STR genotype.
Mycoplasma contamination	The cell line was examined for mycoplasma contamination, and the results were negative. The cell line is our laboratory were passaged on more than 30 times after resuscitation and routinely tested for mycoplasma contamination by PCR.
Commonly misidentified lines (See ICLAC register)	There is no commonly misidentified cell lines in the study.

Animals and other research organisms

Policy information about studies involving animals; ARRIVE guidelines recommended for reporting animal research, and Sex and Gender in Research

Laboratory animals	Three-month-old, 200 g ± 20 g male Sprague Dawley rats (SD rats) were obtained from Laboratory Animal center, Huazhong University of Science and Technology. SD rats were raised under a standard and specific pathogen-free environment with a constant temperature of 21-24 °C and a 1:1 dark:light cycle.
Wild animals	The study didn't involve wild animals.
Reporting on sex	Only male rats participated in this experimental study.
Field-collected samples	The study didn't involve samples collected from the field.
Ethics oversight	Animal obtainment, animal surgical operation and animal sample collection, were approved by Ethics Committee of Tongji Medical College, Huazhong University of Science and Technology (No. S2394).

Note that full information on the approval of the study protocol must also be provided in the manuscript.

Flow Cytometry

Plots

Confirm that:

☒ The axis labels state the marker and fluorochrome used (e.g. CD4-FITC).

☒ The axis scales are clearly visible. Include numbers along axes only for bottom left plot of group (a 'group' is an analysis of identical markers).

☒ All plots are contour plots with outliers or pseudocolor plots.

☒ A numerical value for number of cells or percentage (with statistics) is provided.

Methodology

Sample preparation	1. Cell treatment: after treated according to the experiment arrangement, NP cells were washed twice with PBS, and incubated with labeled EVs or engineered EVs for 72 h,<br>2. Cell preparation: NP cells were washed twice with PBS and digested into cell suspension by using trypsin.
Instrument	FACSCalibur flow cytometer (BD Biosciences, USA)
Software	Collection software: BD CellQuest Pro; analysis software: FlowJo_v10.6.2 and Modfit 5.0
Cell population abundance	The abundance of target cell population in DSC and SSC channels reached more than 90%, and the rest were cell fragments.
Gating strategy	1). Cells in normal group were taken as blank group and delineated by FSC-A and SSC-A channels;<br>2). The cell fluorescence intensity of the normal group was taken as the negative control group;<br>3). In the detection of EGFP or DiO, the positive peak was defined as the cell fluorescence intensity was greater than 3000;<br>4). In the detection of mCherry or PKH26, the positive peak was defined as the cell fluorescence intensity was greater than 2500.

☒ Tick this box to confirm that a figure exemplifying the gating strategy is provided in the Supplementary Information.
